# Biophysical Characterization of the Interaction between a Transport Human Plasma Protein and the 5,10,15,20-Tetra(pyridine-4-yl)porphyrin

**DOI:** 10.3390/molecules27165341

**Published:** 2022-08-22

**Authors:** Otávio Augusto Chaves, Bernardo A. Iglesias, Carlos Serpa

**Affiliations:** 1 CQC-IMS, Department of Chemistry, University of Coimbra, Rua Larga, 3004-535 Coimbra, Portugal; 2 Bioinorganic and Porpyrinoids Materials Lab, Department of Chemistry, Federal University of Santa Maria, Santa Maria 97105-900, Brazil

**Keywords:** porphyrin, human serum albumin, spectroscopy, molecular docking, chemical-biological interactions

## Abstract

The interaction between human serum albumin (HSA) and the non-charged synthetic photosensitizer 5,10,15,20-tetra(pyridine-4-yl)porphyrin (4-TPyP) was evaluated by in vitro assays under physiological conditions using spectroscopic techniques (UV-vis, circular dichroism, steady-state, time-resolved, synchronous, and 3D-fluorescence) combined with in silico calculations by molecular docking. The UV-vis and steady-state fluorescence parameters indicated a ground-state association between HSA and 4-TPyP and the absence of any dynamic fluorescence quenching was confirmed by the same average fluorescence lifetime for HSA without (4.76 ± 0.11 ns) and with 4-TPyP (4.79 ± 0.14 ns). Therefore, the Stern–Volmer quenching (*K_SV_*) constant reflects the binding affinity, indicating a moderate interaction (10^4^ M^−1^) being spontaneous (Δ*G*°= -25.0 kJ/mol at 296 K), enthalpically (Δ*H*° = -9.31 ± 1.34 kJ/mol), and entropically (Δ*S*° = 52.9 ± 4.4 J/molK) driven. Binding causes only a very weak perturbation on the secondary structure of albumin. There is just one main binding site in HSA for 4-TPyP (*n* ≈ 1.0), probably into the subdomain IIA (site I), where the Trp-214 residue can be found. The microenvironment around this fluorophore seems not to be perturbed even with 4-TPyP interacting via hydrogen bonding and van der Waals forces with the amino acid residues in the subdomain IIA.

## 1. Introduction

Human Serum Albumin (HSA) is the most abundant globular protein in the bloodstream (35–50 g/L). It is synthesized in the liver and is responsible for the transport of both endogenous and exogenous compounds, e.g., fatty acids, hormones, metabolites, and commercial drugs to their target [1,2]. For this reason, from a pharmacological point of view, the interaction between HSA and drugs is crucial for a better understanding of both pharmacokinetics and toxicological profiles [3,4]. The structure of HSA has been elucidated by X-ray analysis and was revealed as an ellipsoid with a heart shape consisting of three domains (I, II, and III), and each domain is divided into two subdomains (A and B) [5,6]. Sudlow and coworkers [7] were one of the first researchers to evaluate the specificity of different drugs in binding with albumin and for this reason, the binding sites I and II, located in subdomains IIA and IIIA, respectively, were known as corresponding Sudlow’s sites I and II.

**Figure 1:**
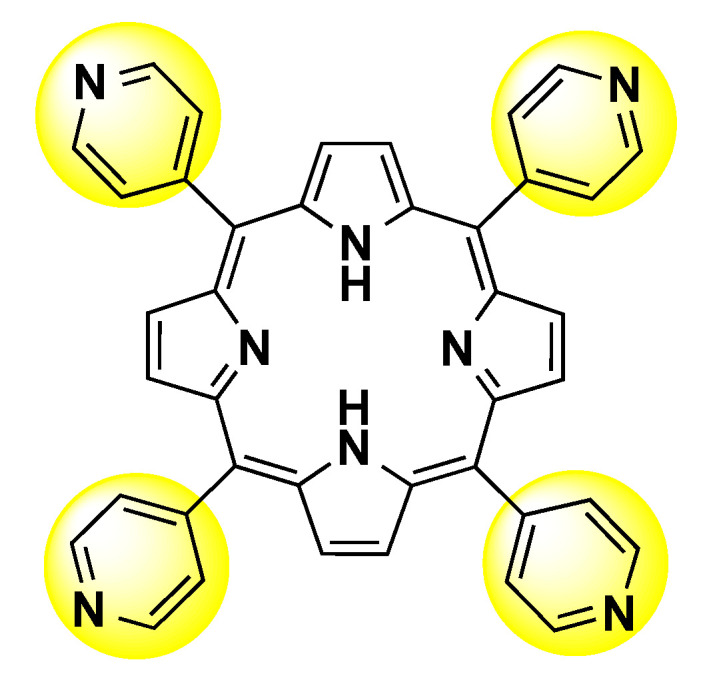
Chemical structure for 5,10,15,20-tetra(pyridine-4-yl)porphyrin.

Porphyrin is a class of compounds containing a flat ring of four linked-heterocyclic groups, sometimes with a central metal atom. There are naturally occurring porphyrins, e.g., protoporphyrin IX, chlorophyll, and cobalamin, however, the synthetic ones have attracted attention mainly due to their applicability as photosensitizers in antimicrobial photodynamic therapy (aPDT) or photodynamic therapy (PDT) of malignant tissues [8,9]. In this sense, 5,10,15,20-tetra(pyridine-4-yl)porphyrin (4-TPyP, Figure 1), a simple non-charged synthetic porphyrin, has attracted attention in the design of novel photosensitizers to PDT, mainly due to its high singlet oxygen quantum yield (Φ_∆_ = 0.76, in acetonitrile) and phototoxicity in a nanomolar scale under green light irradiation (522 nm) with very low light dose (1.0 J/cm^2^) [10]. Recently, binding studies between Bovine Serum Albumin (BSA, a very similar protein compared with HSA, sharing 76% identity and 88% similarity in protein sequence, however, with two tryptophan residues) and 5-phenyl-10,15,20-tri(pyridine-4-yl)porphyrin, a non-charged synthetic porphyrin with structural similarities with 4-TPyP was reported, revealing that the main fluorescence quenching mechanism is static, with binding spontaneous, strong, controlled by electrostatic forces, and the hydrophobicity of the microenvironment around tyrosine (Tyr) and tryptophan (Trp) residues are enhanced in the presence of this porphyrin [11].

Since there are not any biophysical reports on the binding capacity of HSA and 4-TPyP (a relevant porphyrin to develop potential leads for PDT), the present study reports this interaction by multiple spectroscopic techniques (UV-vis, circular dichroism, steady-state, time-resolved, synchronous, and 3D-fluorescence) under physiological conditions at pH = 7.4. To offer a molecular-level explanation of the binding HSA: 4-TPyP, molecular docking calculations were also carried out for the three main binding sites of albumin (subdomains IIA, IIIA, and IB).

## 2. Results

### 2.1. Experimental Binding Capacity of 4-TPyP to HSA

**Figure 2:**
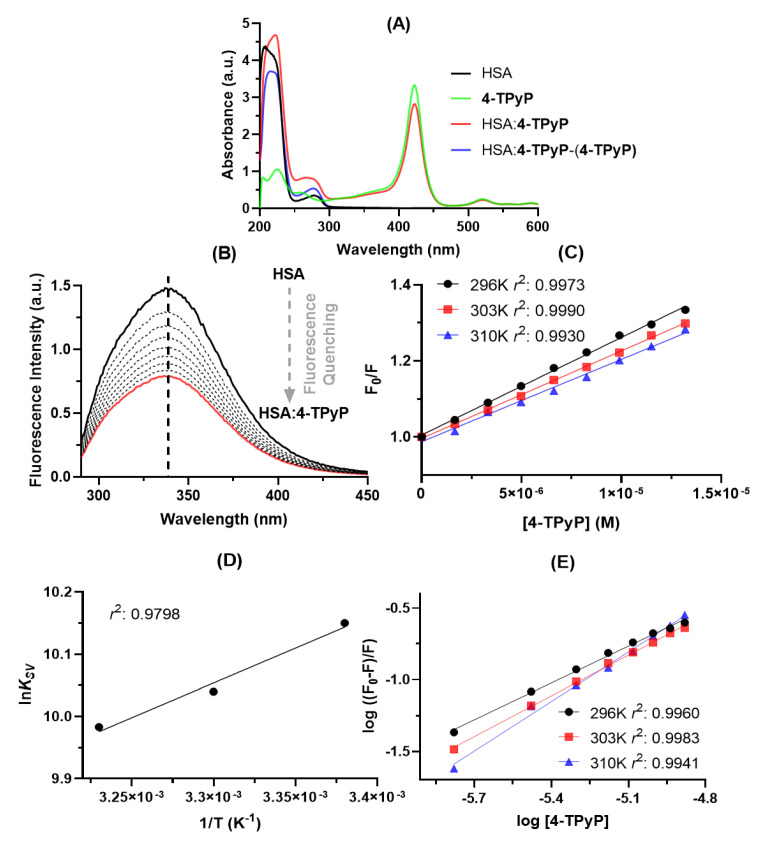
(**A**) UV-vis spectra for HSA (black line, 1.0 × 10^−5^ M), 4-TPyP (green line, 1.32 × 10^−5^ M), HSA:4-TPyP (red line), and mathematical subtraction (HSA:4-TPyP) – (4-TPyP) (blue line) in PBS solution (pH = 7.4) at 310 K. (**B**) Steady-state fluorescence emission spectra for HSA without and upon successive additions of 4-TPyP at 310 K (λ_exc_ = 280 nm). (**C**) Stern–Volmer plots for the interaction HSA:4-TPyP corresponding to the steady-state fluorescence data at three different temperatures. (**D**) Van’t Hoff plot based on *K_SV_* values for HSA:4-TPyP. (**E**) Double logarithmic plots for the interaction HSA:4-TPyP at three different temperatures. The *r*^2^ in each plot is the coefficient of determination. [HSA] = 1.0 × 10^−5^ M and [4-TPyP] = 0.17, 0.33, 0.50, 0.66, 0.83, 0.99, 1.15, and 1.32 × 10^−5^ M.

Absorption spectroscopy is a simple technique to preliminary evaluate qualitatively the binding capacity between HSA and 4-TPyP. From Figure 2A, was noticed that the HSA solution presents two absorption maximums: one at 222 nm due to the π → π* transition of C=O (from amide group) and the other at 280 nm attributed to *n* → π* transition which is associated with the aromatic amino acid residues tryptophan (Trp), phenylalanine (Phe), and tyrosine (Tyr) [3,12]. On the other hand, the absorption peaks at wavelengths higher than 400 nm is only attributed to the electronic transitions of 4-TPyP (Soret and *Q*-bands) [10]. Upon the addition of 4-TPyP in the albumin solution, there is a significant hyperchromic effect in the 250–300 nm range (red line in Figure 2A), however, to assess if the observed hyperchromic displacement is in fact a consequence of the HSA:4-TPyP interaction, and not simply a contribution from porphyrin absorption, the HSA:4-TPyP and 4-TPyP absorption spectra were subtracted. The resulting spectrum in the 250–300 nm range (blue line in Figure 2A) continues to present a hyperchromic effect with a small blue-shift, indicating a ground-state association at this concentration [3,12,13,14]. This phenomenon may be confirmed by the hypochromic effect in the Soret Band (≈400 nm) of 4-TPyP in the presence of HSA. Additionally, there was also evidence of a hyperchromic effect in the 200–250 nm range upon addition of 4-TPyP into the HSA solution, however, after the 4-TPyP absorption spectrum subtraction, this effect was due to the contribution of absorption from 4-TPyP, being the correct effect for this wavelength range as a hypochromic effect that might be considered as preliminary evidence that 4-TPyP can cause some perturbation on the albumin structure [12,15].

Steady-state fluorescence spectroscopy is a more sensitive technique than UV-vis absorption to study the binding capacity of different small compounds with proteins, including porphyrin binding with HSA [14]. Figure 2B depicts the steady-state fluorescence emission of HSA without and upon successive additions of 4-TPyP, indicating that the porphyrin might interact with albumin without perturbing the microenvironment around the fluorophores due to the lack of blue- or red-shift in the maximum emission wavelength [16]. The steady-state fluorescence emission for 4-TPyP was also determined and as expected, there is not any fluorescence emission in the 290–450 nm range (region corresponding to the albumin fluorescence emission).

Table 1 summarizes the binding parameters obtained by steady-state fluorescence data (Figure 2C–E). The Stern–Volmer quenching (*K_SV_*) constant values decrease with increasing temperature and the bimolecular quenching rate (*k_q_*) constant values are 10^12^ M^−1^ s^−1^, being three orders of magnitude larger than the maximum diffusion rate constant in water (*k_diff_* ≈ 7.40 × 10^9^ M^−1^ s^−1^ at 298 K, according to the Smoluchowski–Stokes–Einstein theory at 298 K) [17], indicating a ground-state association between HSA and 4-TPyP (static quenching mechanism) [15], agreeing with UV-vis results.

**Figure 3:**
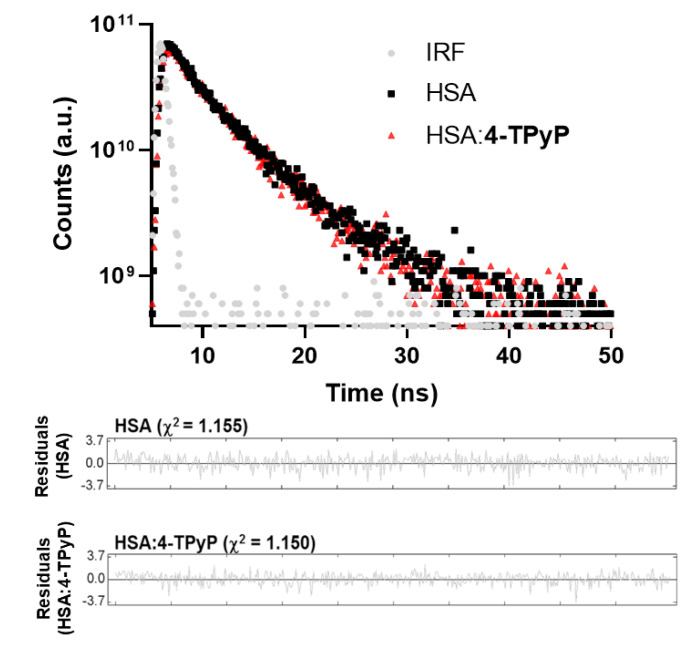
Time-resolved fluorescence decays for HSA without and with 4-TPyP at pH = 7.4 and 296 K using an electrically pumped laser (EPL) with an excitation wavelength of 280 ± 10 nm, pulse width of 850 ps, and a typical average power of 1.8 µW/pulse, monitoring emission at 340 nm. The residuals correspond to the bi-exponential treatment. [HSA] = 1.0 × 10^−5^ M and [4-TPyP] = 1.32 × 10^−5^ M.

To further confirm the main fluorescence quenching mechanism detected by both UV-vis analysis and Stern–Volmer approximation, time-resolved fluorescence decays were obtained without and with the maximum porphyrin concentration used in the UV-vis absorption and steady-state fluorescence (Figure 3). The HSA decay without porphyrin showed two fluorescence lifetimes: τ_1_ = 1.67 ± 0.13 and τ_2_ = 5.67 ± 0.11 ns, with relative percentage of 22.7% and 77.3%, respectively, agreeing with the literature [18,19], while the complex HSA:4-TPyP showed τ_1_ = 1.80 ± 0.19 and τ_2_ = 5.52 ± 0.13, with a relative percentage of 19.7% and 80.3%, respectively. Amiri and coworkers [20] observed that the fluorescence decay for emissive amino acid Trp in HSA yields three lifetimes. The first two lifetimes are already observed for free-Trp (attributed to excited-state Trp substructures) and the third, longer one, results from interactions between the Trp residue and its microenvironment in the protein matrix. The first lifetime is short (sub nanosecond) with a low pre-exponential factor (3%). Thus, due to the instrumental limitations, most reports (as we in the present work) observe solely two lifetimes. Values of the two fluorescence lifetimes and of their pre-exponentials (this is, the relative population of the Trp excited states which are the origin of the fluorescence) may depend on the microenvironment and on the extension and nature of interactions formed upon HAS: ligand complex formation. The measured lifetimes values and the respective percentages do not vary significantly with the formation of the complex, evidence that the microenvironment around the fluorophore is not perturbed very much with the presence of 4-TPyP.

Since the average fluorescence lifetime of HSA without (4.76 ± 0.11 ns) and with 4-TPyP (4.79 ± 0.14 ns) is the same inside the experimental error, time-resolved fluorescence analysis indicates the absence of dynamic quenching mechanisms and confirmed the ground-state association between HSA and 4-TPyP [15,21]. Therefore, the *K_SV_* values can also estimate the binding affinity [15,22], being in the order of 10^4^ M^−1^. For this reason, the double-logarithmic approximation was applied only to determine the number of binding sites (*n*) in the range of 0.857–1.15, which indicates an interaction in the proportion 1:1—one single albumin molecule binds with one molecule of 4-TPyP [23].

The negative Δ*G*° values are consistent with the spontaneity of the binding process in all the evaluated temperatures and since Δ*H*° and Δ*S*° values are negative and positive, respectively, the association HSA:4-TPyP is enthalpically and entropically driven [24].

### 2.2. Structural and Microenvironment Perturbation of HSA upon 4-TPyP Binding

**Figure 4:**
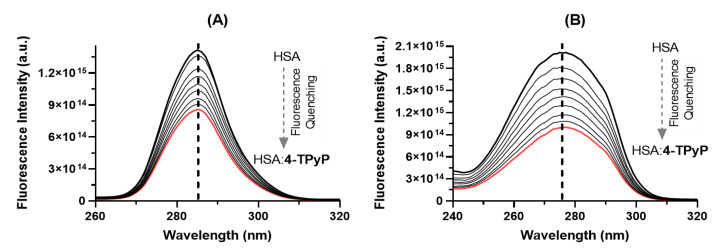
The SF spectra of HSA without and upon successive additions of 4-TPyP at (**A**) Δλ = 15 nm and (**B**) Δλ = 60 nm in pH = 7.4 at room temperature. [HSA] = 1.0 × 10^−5^ M and [4-TPyP] = 0.17, 0.33, 0.50, 0.66, 0.83, 0.99, 1.15, and 1.32 × 10^−5^ M.

Comparing the steady-state fluorescence technique with the synchronous fluorescence (SF), the last one has been considered a complementary and more sensitive approach to detect possible perturbations in the microenvironment around the two main fluorophores of albumin (Tyr and Trp residues) after drug binding [25,26,27]. Figure 4 shows the SF spectra for HSA without and upon successive additions of 4-TPyP at Δλ 15 and 60 nm for Tyr and Trp residues, respectively. For both Δλ, there is a significant decrease in the fluorescence signal upon additions of porphyrin, however, it did not induce any blue- or red-shift, agreeing with steady-state fluorescence data (Figure 2B), which indicated that the binding of 4-TPyP does not induce any significant perturbation on the microenvironment around the fluorophores.

**Figure 5:**
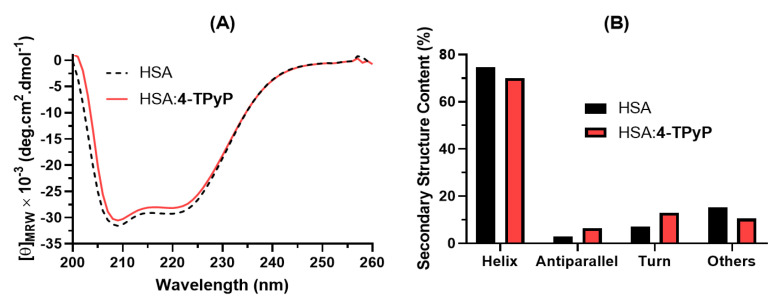
(**A**) Far-UV CD spectra for HSA without and with 4-TPyP in PBS (pH 7.4) at 310K. (**B**) Secondary structure content for HSA without and with 4-TPyP determined by the online server BestSel (Beta Structure Selection http://bestsel.elte.hu/index.php (accessed on 2 June 2022)). [HSA] = 1.0 × 10^−6^ M and [4-TPyP] = 1.32 × 10^−5^ M.

Circular dichroism (CD) plays an essential role in studying perturbation on the secondary structure of albumin upon drug binding [28,29]. Figure 5A depicts the CD spectra for HSA without and with 4-TPyP, while Figure 5B shows the corresponding secondary structure content. The CD spectra of HSA and HSA:4-TPyP are practically similar in shape and peak position: two minimum peaks, one at 208 nm and the other at 222 nm. Additionally, the secondary structure content did not differ significantly even upon the addition of 4-TPyP in a proportion HSA:4-TPyP of almost 1:13.

**Figure 6:**
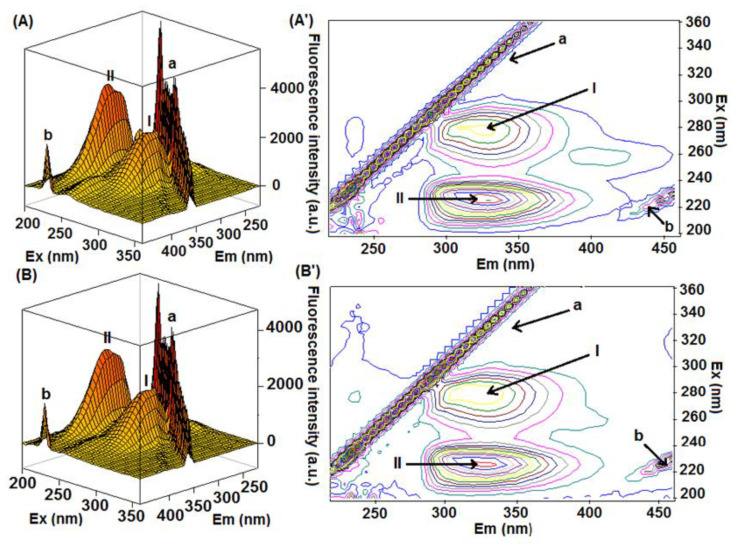
The 3D-fluorescence spectra and the corresponding contour maps for (**A**,**A’**) HSA and (**B**,**B’**) HSA:4-TPyP in pH = 7.4 at room temperature. [HSA] = 1.0 × 10^−6^ M and [4-TPyP] = 1.32 × 10^−6^ M.

Finally, the 3D-fluorescence spectroscopy was applied as additional and conclusive evidence for the experimental data obtained by steady-state fluorescence, SF, and CD spectra [30]. Figure 6 depicts the 3D-fluorescence spectra of HSA and HSA:4-TPyP and their corresponding contour maps, while Table 2 summarizes the fluorescence characteristics of these spectra. Peaks I (λ_ex_ = 280 nm) and II (λ_ex_ = 225 nm) are characteristics of the intrinsic fluorescence spectral behavior of HSA, mainly due to the absorption of the fluorophores Tyr and Trp, however, the peaks “a” (λ_ex_ = λ_em_) and “b” (2λ_ex_ = λ_em_) are characteristics of Rayleigh and second-order scattering, respectively [31,32]. The presence of 4-TPyP in the HSA solution did not cause a significant Stokes shift in the position of peaks I and II, as well as the fluorescence intensity was reduced by 10.8% and 16.6% for peaks I and II, respectively, reinforcing the data obtained by the other spectroscopic techniques. It is important to highlight that in this technique, the scattering peaks are so high probably due to the aggregation of 4-TPyP in the PBS medium, indicating that despite the presence of HSA, the non-charged porphyrin 4-TPyP still can form some aggregate.

### 2.3. Molecular-Level Explanation on the Binding Capacity of 4-TPyP to HSA

**Figure 7:**
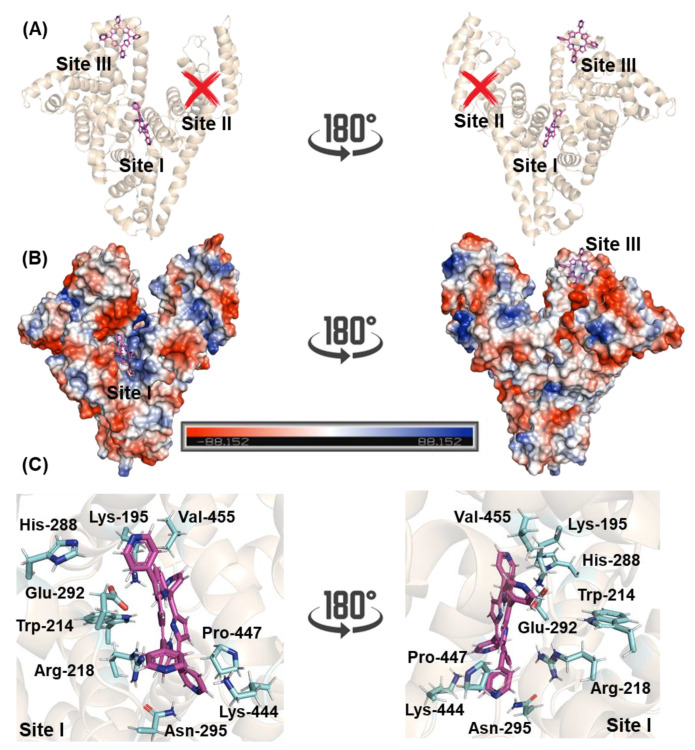
Superposition of molecular docking results for the interaction HSA:4-TPyP into subdomains IIA and IB (sites I and III, respectively) in terms of (**A**) cartoon representation and (**B**) electrostatic potential map for albumin (blue and red for positive and negative electrostatic density, respectively). (**C**) The main amino acid residues that interact with 4-TPyP into site I. Selected amino acid residues and 4-TPyP are represented as sticks in cyan and pink, respectively. Elements’ color: hydrogen, nitrogen, and oxygen in white, dark blue, and red, respectively.

The 3D structure of HSA has three main binding sites with different specificities: site I, also known as Sudlow’s site I, located in the subdomain IIA, site II, also known as Sudlow’s site II, located in the subdomain IIIA, and site III located in the subdomain IB [6,33,34]. To suggest the main binding site and to offer a molecular-level explanation of the binding capacity of 4-TPyP to HSA, molecular docking calculations were carried out. The docking score value (dimensionless) for sites I and III was 51.5 and 45.7, respectively, while 4-TPyP did not have any favorable pose for site II. Figure 7A,B depict the superposition of the binding pose of 4-TPyP into subdomains IIA and IB in a cartoon and electrostatic representation, respectively. The 4-TPyP interacts preferentially in a positive electron density pocket being stabilized mainly by hydrogen bonding and van der Waals forces with the amino acid residues of albumin (Figure 7C and Table 3), i.e., not only the main albumin’s fluorophore Trp-214 residue interacts with the pyridyl moiety of 4-TPyP via van der Waals forces within a distance of 3.50 Å, but also the amino acid residues His-288, Lys-444, Pro-447, and Val-455 interact by the same intermolecular force (van der Waals) with the pyridyl moieties of 4-TPyP structure within a distance of 3.10, 2.90, 3.70, and 3.20 Å, respectively. On the other hand, the hydrogen atom from the polar group of Lys-195, Arg-218, and Asn-295 is potential donor for hydrogen bonding with 4-TPyP within a distance of 3.40, 3.40, and 3.30 Å, respectively, while the negative charged carboxyl group of Glu-292 is a potential acceptor for hydrogen bonding with the amino group of the tetrapyrrolic core from 4-TPyP structure within distance of 3.40 Å. Finally, molecular docking calculations did not suggest any π-π, π-alkyl, or non-conventional hydrogen bonding interactions between 4-TPyP and the amino acid residues into subdomain IIA.

## 3. Discussion

The interaction between non-charged or charged porphyrins to albumin has been widely reported as a hot topic by different researchers, however, these studies do not apply the same mathematical approximations and/or methodologies among them [11,14,22,34,35,36,37,38,39,40], making a direct comparison with our data difficult. In this sense, for a better interpretation of the binding capacity between HSA and 4-TPyP, reports were selected for porphyrins with both similar structure and experimental/mathematical approaches, more specifically with a non-charged porphyrin (5-phenyl-10,15,20-tri(pyridine-4-yl)-porphyrin, TriPyP) [11] and charged positively porphyrins 5,10,15,20-tetrakis(4-1-benzylpyridinium-4-yl)porphyrin (TBzPyP) [14], 5,10,15,20-tetrakis(1-[Ru(bpy)_2_Cl]-pyridinium-4-yl)porphyrin (4-RuTPyP) [38], 5,10,15,20-tetrakis(1-methyl-pyridinium-4-porphyrin (4-TMPyP), and 5,10,15,20-tetrakis(1-[Pt(bpy)Cl]-pyridinium-4-yl)porphyrin (4-PtTPyP) [22].

The preliminary binding evaluation was carried out by UV-vis absorption technique, demonstrating hypochromic and hyperchromic effects in the 200–250 and 250–300 nm range, respectively, after the subtraction of the 4-TPyP absorption signal in the UV-vis absorption spectrum of HSA:4-TPyP, indicating that 4-TPyP can cause some perturbation on the albumin structure (analysis in the 200–250 nm range) and there is a ground-state association for HSA:4-TPyP (analysis in the 250–300 nm range) [12,13]. Unfortunately, we did not find any reports for the binding albumin:porphyrin under the same UV-vis approach that we conducted (subtraction of porphyrin absorption signal in the UV-vis absorption profile of albumin:porphyrin) to compare our data with similar porphyrin structures.

The binding of 4-TPyP to HSA does not cause any shift in the maximum fluorescence peak of albumin by both steady-state, synchronous, and 3D-fluorescence techniques, being a clear indication that the evaluated non-charged porphyrin does not perturb the microenvironment around the main fluorophores (Trp, Tyr, and Phe), having the same trend compared to TriPyP [11] and 4-TMPyP [22], however, a different trend considering 4-PtTPyP [22] and 4-RuTPyP [38], indicating that the positive charge in porphyrin structure is not a crucial step in perturbing the microenvironment around the fluorophores, but there is a significant dependence of the steric volume of the groups covalently connected with pyridyl moiety.

The ground-state association for HSA:4-TPyP previously detected by UV-vis analysis was reinforced by both steady-state and time-resolved fluorescence data, showing that the fluorescence quenching mechanism of HSA by 4-TPyP is purely static. Therefore, the double-logarithmic approximation to obtain the *K_a_* values (0.0405, 0.101, and 1.19 × 10^5^ M^−1^ at 296, 303, and 310 K, respectively) is not the best mathematical treatment to estimate the binding affinity of HSA:4-TPyP, reinforcing that in this case, the *K_SV_* values besides evaluating the quenching mechanism also determine the binding affinity [15]. As a drug carrier, HSA may aid in the selective delivery of porphyrins to a tumor region and facilitate drug access into the cell via receptor mechanisms (moderate binding affinity for HSA). On the other hand, the same carrier may cause a decrease in the amount of porphyrin available for PDT by its rapid removal from circulation (strong or weak binding affinity for HSA) [37,41]. Since the *K_SV_* values are in the order of 10^4^ M^−1^, 4-TPyP binds moderately with albumin, which is favorable to achieving the ideal pharmacokinetic profile for PDT. The same trend was identified in 4-TMPyP and 4-PtTPyP [22], while the porphyrins TBzPyP [14], TriPyP [11], and 4-RuTPyP [38] bind stronger with albumin, indicating that there is not necessarily a charge or steric volume dependence on the binding affinity of pyridyl-porphyrins to albumin but there is a crucial dependence on the nature of the chemical group connected covalently with pyridyl moiety.

In all evaluated temperatures, negative Δ*G*° values were obtained, which are consistent with the spontaneity of the binding HSA:4-TPyP and since there are negative and positive values for Δ*H*° and Δ*S*°, respectively, both thermodynamics parameters contribute to the negative Δ*G*° value, therefore, the association HSA:4-TPyP is considered enthalpically and entropically driven. According to Ross and Subramanian [42] Δ*H*° < 0 and Δ*S*° > 0, indicate that electrostatic forces might contribute significantly to the complex stability, agreeing with in silico data which detected that besides hydrogen bonding, the electrostatic van der Waals interactions play a key intermolecular force for the interaction HSA:4-TPyP into subdomain IIA (site I). Additionally, Δ*S*° > 0 can also be correlated with the hydrophobic effect governed by desolvation factors upon binding of 4-TPyP into HSA [43]. Interestingly, these results are opposite from those reported for the charged porphyrins 4-RuTPyP [38], 4-TMPyP, and 4-PtTPyP [22] that identified subdomain IB (site III) as the main binding region, possibly due to the negative electrostatic potential surface of subdomain IB. Unfortunately, there are no in silico or experimental data to identify the main binding site of TBzPyP [14] and TriPyP [11], however, the thermodynamics parameters of TriPyP [11] are like those obtained of 4-TPyP, indicating that the non-charged pyridyl-porphyrins derivatives might interact by the same types of intermolecular forces.

## 4. Materials and Methods

### 4.1. Chemicals and Instruments

Commercially available phosphate buffer solution (PBS) and HSA (lyophilized powder, fatty acid-free, globulin free with purity higher than 99%, code A3782-1G) were obtained from Sigma-Aldrich/Merck (St. Louis, MO, USA). One tablet of PBS dissolved in 200 mL of millipore water yields a 0.01 M, 0.0027 M, and 0.137 M of phosphate buffer, potassium chloride, and sodium chloride, respectively, with pH 7.4 at 298 k. Water used in all experiments was Millipore water. Acetonitrile (spectroscopic grade) was obtained from Vetec (Rio de Janeiro, Brazil). The porphyrin 4-TPyP was purchased from PorphyChem (Dijon, France) and a stock solution was prepared in acetonitrile. To increase the solubility of porphyrin in the stock solution the mixture was heated to 323 K and inserted into a home-built ultrasound system. The chemical stability of 4-TPyP under this condition was determined following its corresponding UV-vis profile [10].

The UV-vis, steady-state fluorescence, and circular dichroism (CD) spectra were measured on a Jasco model J-815 optical spectrometer (Easton, MD, USA) and a thermostatic cuvette holder Jasco PFD-425S15F (Easton, MD, USA) was applied to control the temperature in the quartz cell (1.00 cm optical path). All spectra were obtained as the average of three scans with appropriate background corrections. Time-resolved fluorescence measurements were performed on an Edinburgh Instruments fluorimeter model FL920 CD (Edinburgh, UK), equipped with an EPL with excitation wavelength of 280 ± 10 nm, pulse width of 850 ps, and a typical average power of 1.8 µW/pulse, monitoring emission at 340 nm. Synchronous fluorescence (SF) and 3D-fluorescence spectra were performed by the Edinburgh Instruments fluorimeter model Xe900 (Edinburgh, UK).

### 4.2. Spectroscopic Measurements

The UV-vis spectra were obtained in three different conditions in a quartz cell with 1.00 cm optical path: HSA solution (1.0 × 10^−5^ M in PBS), 4-TPyP solution (1.32 × 10^−5^ M in PBS), and HSA:4-TPyP mixture at 310 K in the 200-600 nm range.

The steady-state fluorescence measurements in the 290-450 nm range (λ_exc_ = 280 nm, was used the excitation wavelength of 280 nm instead of 295 nm due to the highest absorption contribution of albumin than porphyrin to minimize the inner filter effect) were carried out for 3.0 mL of HSA solution (1.0 × 10^−5^ M, in PBS), without and with 4-TPyP (added manually by a micro syringe to achieve final concentrations of 0.17, 0.33, 0.50, 0.66, 0.83, 0.99, 1.15, and 1.32 × 10^−5^ M) at 296, 303, and 310 K. The maximum concentration of 4-TPyP used in the binding assays corresponds to a stock aliquot of 40 μL of acetonitrile that does not perturb both protein structure and spectroscopic signal [38]. To compensate for the inner filter effect, the maximum steady-state fluorescence intensity values for the system HSA:4-TPyP were corrected by the absorption of porphyrin at excitation (λ = 280 nm) and emission wavelengths (λ = 340 nm), applying Equation (1) [43,44]:

(1) $$F_{cor}=F_{obs}10^{\left[\frac{\left(A_{ex}+A_{em}\right)}{2}\right]}$$

where *F_cor_* and *F_obs_* are the corrected and observed steady-state fluorescence intensity values, respectively, while *A_ex_* and *A_em_* are the absorption value at the excitation (λ = 280 nm, ε = 7537.3 M^−1^cm^−1^) and maximum fluorescence emission (λ = 340 nm, ε = 12,659.3 M^−1^cm^−1^) wavelengths, respectively.

To obtain quantitative parameters on the binding capacity of HSA:4-TPyP, the maximum fluorescence data after inner filter correction were treated by Stern–Volmer (Equation (2)), double-logarithmic (Equation (3)), van’t Hoff (Equation (4)), and Gibbs’ free energy (Equation (5)) approximations [15,45,46,47].

(2) $$\frac{F_{0}}{F}=1+k_{q}\tau_{0}\;\left[Q\right]=1+K_{SV}\left[Q\right]$$

(3) $$\log\;\left(\frac{F_{0}-F}{F}\right)=n\;\log\;\left[Q\right]+\log K_{a}$$

(4) $$\ln K_{SV}=-\frac{\Delta H{}^{\circ}}{RT}+\frac{\Delta S{}^{\circ}}{R}$$

(5) ΔG°= ΔH°−TΔS°

where *F*_0_ and *F* are the steady-state fluorescence intensities of HSA without and with 4-TPyP, respectively. The [*Q*], *K_SV_*, and *k_q_* are the porphyrin concentration, Stern–Volmer quenching constant, and bimolecular quenching rate constant, respectively. The τ_0_ is the obtained experimental average fluorescence lifetime for HSA without 4-TPyP in PBS (τ_0_ = 4.76 ± 0.11 ns), while *K_a_* and *n* are the binding constant and number of binding sites, respectively. The Δ*H*°, Δ*S*°, and Δ*G*° are the enthalpy, entropy, and Gibbs’ free energy change, respectively. Finally, *T* and *R* are the temperature (296, 303, and 310 K) and gas constant (8.3145 Jmol^−1^K^−1^), respectively.

Time-resolved fluorescence decays were obtained for 3.0 mL of HSA solution (1.0 × 10^−5^ M, in PBS) without and with 4-TPyP (1.32 × 10^−5^ M) at room temperature. The instrumental response function (IRF) was collected using a Ludox^®^ dispersion.

The SF spectra were obtained for 3.0 mL of has solution (1.0 × 10^−5^ M, in PBS) without and upon successive additions of 4-TPyP in the same concentrations of porphyrin used in the steady-state fluorescence studies (0.17, 0.33, 0.50, 0.66, 0.83, 0.99, 1.15, and 1.32 × 10^−5^ M) at room temperature in the range of 260-320 nm for Tyr (Δλ = 15 nm) and 240-320 nm for Trp (Δλ = 60 nm). Finally, the 3D-fluorescence spectra were recorded at the 240–320 nm range for 3.0 mL of HSA solution (1.0 × 10^−6^ M, in PBS) without and upon addition of 4-TPyP (1.32 × 10^−6^ M) using λ_exc_ = 200–360 nm and λ_em_ = 200–460 nm, at room temperature.

The CD spectra (200–260 nm) were recorded for HSA without and with 4-TPyP in PBS at 310 K. The HSA concentration was fixed at 1.0 × 10^−6^ M and porphyrin concentration was those to achieve proportion HSA:4-TPyP of 1:13. The average spectra were obtained from three successive runs and corrected by subtraction of the buffer signal. The CD raw data in ellipticity (Θ_obs_, in millidegrees) were normalized and expressed as the mean residue weight ([Θ]_MRW_) in deg. cm^2^-dmol^−1^, defined as [Θ]_MRW_ = (Θ_obs_ × 10^−3^) × 100 × MW/(*l* × *c* × NAA), where MW is the protein molecular weight, *c* is the protein concentration in milligrams per milliliter, *l* is the light path length in centimeters, and NAA is the number of amino acids per protein. The secondary structure content was estimated by analysis of the CD spectra using the online server BestSel (Beta Structure Selection http://bestsel.elte.hu/index.php (accessed on 2 June 2022)).

### 4.3. Molecular Docking Procedure

The 4-TPyP structure was built and energy-minimized by the Density Functional Theory (DFT) method with the potential B3LYP and basis set 6-31G*, available in the Spartan’14 software (Wavefunction, Inc., Irvine, USA). The crystallographic structure of HSA was obtained in the Protein Data Bank (PDB), with access code 3JRY [48]. Molecular docking calculations were performed with GOLD 2020.2 software (CCDC, Cambridge Crystallographic Data Centre, Cambridge, UK).

Hydrogen atoms were added to HSA according to the data inferred by GOLD 2020.2 software on the ionization and tautomeric states at pH 7.4. Docking interaction cavity in the protein was established with 8 Å radius from the amino acid residues Trp-214, Tyr-411, and Tyr-161 for sites I, II, and III, respectively. The number of genetic operations (crossover, migration, mutation) in each docking run was set to 100,000. The scoring function used was ‘*ChemPLP*’, which is the default function of GOLD 2020.2 software. Figures for the docking poses were generated using PyMOL Delano Scientific LLC software (Schrödinger, New York, NY, USA).

## Figures and Tables

**Table 1:** Binding parameters for the interaction HSA:4-TPyP in PBS (pH = 7.4) at three different temperatures.

T (K)	*K_SV_* × 10^4^ (M^−1^) ^[a]^	*k_q_* × 10^12^ (M^−1^s^−1^) ^[a]^	*n* ^[b]^	Δ*H°* (kJ/mol) ^[c]^	Δ*S°* (J/molK) ^[c]^	Δ*G°* (kJ/mol)
296	2.57 ± 0.05	5.39 ± 0.11	0.857 ± 0.022	−9.31 ± 1.34	52.9 ± 4.4	−25.0
303	2.30 ± 0.03	4.84 ± 0.06	0.948 ± 0.016			−25.3
310	2.17 ± 0.07	4.55 ± 0.14	1.15 ± 0.04			−25.7

^[a]^ Corresponding to Stern–Volmer plots, ^[b]^ Corresponding to double-logarithmic plots, ^[c]^ Corresponding to van’t Hoff plot.

**Table 2:** The 3D-fluorescence spectral characteristics for HSA and HSA:4-TPyP in pH = 7.4 at room temperature.

System	Peak	Peak Position (λ_exc_/λ_em_ nm/nm)	Intensity × 10^3^ (a.u.)
	a	225/225 → 355/355	4.00 → 1.89
HSA	b	230/460	2.01
	I	280/335	2.13
	II	225/330	3.56
	a	225/225 → 355/355	3.16 → 1.66
HSA:4-TPyP	b	230/460	1.66
	I	280/340	1.90
	II	225/335	2.97

**Table 3:** The main amino acid residues and interactive forces for HSA:4-TPyP in the site I.

Amino Acid Residue	Interaction	Distance (Å)
Lys-195	Hydrogen bonding	3.40
Trp-214	Van der Waals	3.50
Arg-218	Hydrogen bonding	3.40
His-288	Van der Waals	3.10
Glu-292	Hydrogen bonding	3.40
Asn-295	Hydrogen bonding	3.30
Lys-444	Van der Waals	2.90
Pro-447	Van der Waals	3.70
Val-455	Van der Waals	3.20

## Data Availability

All analyzed data are contained in the main text of the article. Raw data are available from the authors upon request.
